# New Advances and Current Trends in Bioavailability of Coffee and Coffee By-Product Constituents and Their Metabolites: A Review

**DOI:** 10.3390/ijms27219841

**Published:** 2026-11-03

**Authors:** Boris Nemzer, Fadwa Al-Taher, Nebiyu Abshiru, Elvira de Mejia

**Affiliations:** 1 VDF FutureCeuticals, Inc., Momence, IL 60954, USA; bnemzer@futureceuticals.com (B.N.); fadwa.al-taher@futureceuticals.com (F.A.-T.); nebiyu.abshiru@futureceuticals.com (N.A.); 2 228 ER Madigan Laboratory, Department of Food Science and Human Nutrition, University of Illinois, Urbana-Champaign, 1201 W. Gregory Dr., Urbana, IL 61801, USA

**Keywords:** bioactives, bioavailability, caffeine, chlorogenic acids, coffee, delivery systems

## Abstract

Coffee is one of the most widely consumed beverages worldwide, yet understanding how its bioactive compounds are absorbed and exert health effects remains incomplete. The objective and scope of this review is to critically summarize current advances in coffee bioavailability research, identify major limitations, and highlight emerging research trends. Recent research has advanced in three core areas. First, genetic stratification has moved the field beyond one-size-fits-all approaches by genotyping for key polymorphisms to explain inter-individual variability. Second, advanced metabolomics, including targeted and untargeted LC-MS/MS and multi-omics integration, has provided comprehensive views of phenolic absorption and bioavailability. Third, rigorous intervention designs, including randomized crossover trials and large prospective cohorts with detailed exposure assessment, have improved causal inference. Despite these advances, major limitations persist. Many studies lack sufficient statistical power to detect gene-treatment interactions. The clinical relevance of discovered metabolites is often not established. Observational cohorts remain susceptible to residual confounding, and the field lacks a standardized food matrix for cross-study comparisons. Key strategies to improve the bioavailability of coffee and its by-products were proposed, including novel delivery systems for coffee phytochemicals, human clinical trials involving encapsulated coffee bioactive compounds, and exploring coffee by-products as novel bioactive sources.

## 1. Introduction

Coffee is a beverage enjoyed by many worldwide for its pleasant aroma and taste. In 2025, over 169 million 60 kg bags of coffee were consumed globally, with the European Union (23.7%), the United States (15.1%), and Brazil (13.2%) the primary consumers [1]. In June 2025, global green bean exports increased 3.3% to 10.23 million bags, up from 9.91 million bags in June 2024. Roasted bean exports increased by 58.1% in June 2025, reaching 0.08 million bags, compared to 0.05 million bags in June 2024 [2]. Consequently, the coffee industry generates large amounts of waste and by-products, accounting for approximately 50% of the fruit’s dry weight. These include pulp (consists of most of the whole coffee cherry fruit that contains the two seeds, coffee beans), coffee husk (CH; the outer skin of the whole coffee cherry), and coffee silverskin (CS; the thin skin that covers the coffee bean), as well as spent coffee grounds (SCG), the primary residual by-product of coffee beverage preparation [3,4].

Coffee contains various bioactive compounds, the most noteworthy of which are caffeine, trigonelline, chlorogenic acids (CGAs), diterpenes, and melanoidin, all of which contribute to its aroma and health benefits. The phytochemical levels depend on the coffee species (Arabica or Robusta), the degree of roasting, and the brewing method [5]. Moreover, coffee by-products have recently been recognized as valuable sources of bioactive compounds and share a phytochemical profile like that of coffee [3,6,7]. Coffee consumption may help prevent inflammatory and oxidative stress-related diseases, such as obesity, metabolic syndrome, and type 2 diabetes, and it is also associated with a lower prevalence of several types of cancer [8,9]. Ingesting coffee in moderation can help prevent and delay the onset of neurodegenerative diseases [10,11]. The European Food Safety Authority (EFSA) established the caffeine intake limit at a safe level to be 400 mg/day (about 5.7 mg/day body weight) in a single dose for healthy adults, and 200 mg/day (about 3 mg/kg body weight) for children, adolescents, and pregnant women [12]. The U.S. Food and Drug Administration (FDA) states that consuming up to 400.0 mg of caffeine per day, corresponding to about four cups of coffee, is not a safety concern [8,13].

Grundy et al. (2025) [14] define bioaccessibility as the portion of an ingested compound that is released from the food matrix and solubilized in gastrointestinal fluids, thereby becoming absorbable. Bioactive compounds need to be bioaccessible to exert their intended health effects within target organs. In contrast, bioavailability is determined by four sequential steps: absorption, distribution, metabolism, and excretion (ADME) [15]. Bioavailability refers to the proportion of a nutrient that is first digested, then absorbed, and finally available at the site of action. This parameter indicates the amount of the active compound that enters the bloodstream to produce a therapeutic effect [16,17]. If a compound does not reach the site of action at sufficient concentration, it remains ineffective. In contrast, successful interaction with the target can induce physiological effects such as increased blood flow, vasodilation, altered cell signaling, and modulation of glucose levels [17].

This review focuses on understanding new advances and current trends of the gastrointestinal fate of coffee and its by-products and their bioactive compounds through human intervention studies, and on investigating novel strategies to improve their bioefficacy and highlight their health-promoting effects. Current advancements include the development of novel delivery methods to enhance the bioaccessibility and bioavailability of beneficial coffee compounds.

## 2. Phytochemical Characterization of Coffee and Coffee By-Product Constituents and Their Potential Health Effects

This section examines the principal phytochemicals and nutrients present in coffee and its by-products that affect aroma, bioactivity, and potential health benefits.

### 2.1. Caffeine

Caffeine is the most abundant alkaloid in coffee. Caffeine is thermally stable, and its content does not change significantly during roasting; however, it may be lower due to sublimation [18,19]. The caffeine content varies among coffee brews prepared using different methods. The caffeine content of the most common brews, expressed in mg/100 mL, is as follows: Espresso (253.4), Turkish coffee (166.5), Moka (152.4), boiled coffee (126.5), and soluble coffee (132.0) [18]. Caffeine concentration varied in the two by-products of coffee processing, CS and SCG, from 27,365 to 41,877 µg/g dry weight and from 32,838 to 54,440 µg/g dry weight, respectively, depending on the extraction solvent [20]. Caffeine stimulates brain function, improves mood, and enhances physical performance. Caffeine is one of the compounds in coffee responsible for reducing the risk of the development of chronic degenerative diseases such as Parkinson’s and Alzheimer’s diseases, and death [18]. Caffeine is associated with antioxidant, anti-inflammatory, and antimicrobial activities. Also, caffeine, often combined with other bioactive compounds such as chlorogenic acids and trigonelline, has been reported to contribute to coffee’s hepatoprotective effects [18].

### 2.2. Trigonelline

Trigonelline, a bioactive pyridine alkaloid, is the second-most abundant alkaloid in green coffee beans after caffeine, with concentrations of 0.2–7.2 g/kg, and coffee by-products (i.e., coffee leaves, flowers, CH or pulp, parchment, CS, and SCG) of 0.4–62.6 g/kg [21]. Trigonelline degrades during the roasting of coffee beans to its nutritional precursor, nicotinic acid (niacin or Vitamin B_3_), as well as volatile coffee constituents such as pyridines, pyrrole derivatives, methyl esters of nicotinic acid, and other compounds responsible for the bitterness and aroma in coffee. Trigonelline has antimicrobial, anticarcinogenic, antioxidative, antilipidemic, hypocholesterolemic, and hypoglycemic effects. It also has the potential to improve memory and exhibit neuroprotective activity. Moreover, it has a protective effect against kidney stone formation [21,22].

### 2.3. Chlorogenic Acids

Chlorogenic acids (CGAs) are the major polyphenolic compounds in coffee, comprising 12–18% of the dry weight of green coffee [5,23]. When coffee is roasted, chlorogenic acids break down, forming lactones and phenolic derivatives, such as caffeic acids, which further hydrolyze, imparting bitterness and astringency to the coffee [23]. CGA levels can range from 16.0 to 400.0 mg per cup in espresso coffee and from 60.0 to 420.0 mg per cup in French press brews [24].

SCG and CS are good sources of chlorogenic acids. Zengin et al. (2020) [20] reported CGA levels of 7.6–14.1 mg/g dry weight in SCG and 4.3–8.9 mg/g dry weight in CS, depending on the extraction solvent. The most abundant CGAs were 5-caffeoylquinic acid, followed by 3-caffeoylquinic acid [19]. Iriondo-DeHond et al. (2019) [25] found that the CS contained high levels of the major CGA, 5-caffeoylquinic acid (9.4 mg/g in Arabica coffee and 21.3 mg/g in Robusta). CGAs have numerous beneficial health effects, including protection against neurodegenerative disorders and diabetic peripheral neuropathy; anti-inflammatory effects; antioxidant properties; antimicrobial activities; reductions in cardiovascular disorders, skin diseases, diabetes mellitus, liver, and kidney injuries; and anti-tumor activities [26].

### 2.4. Diterpenes

Lipids are essential for the viscosity of the coffee brew, foam stability, and flavor retention. The main coffee diterpenes found in the lipid fraction of coffee beans and brew are cafestol, kahweol, and 16-*O*-methylcafestol, which is lower but stable [5,27,28]. Brand et al. (2024) [29] demonstrated that boiled coffee brew contains 127.5–132.7 mg/L of cafestol, which varies with brewing method, roasting degree, grain size, cooking time, and grain-to-water ratio. Lipids and lipophilic coffee compounds such as fatty esters of the diterpenes kahweol and cafestol, and coffee wax constituents, i.e., 5-hydroxytryptamides, remain in the filter, while the hydrophilic compounds pass [30]. Ren et al. (2019) [31] determined that unfiltered coffee contains 3–6 mg per cup of each diterpene.

Wu et al. (2022) [28] observed that diterpenes have both beneficial and unfavorable effects on human health. Diterpenes exhibit free radical-inhibitory properties that may contribute to cancer prevention. However, excessive intake can raise total cholesterol, blood lipids, and homocysteine levels, which may lead to cardiovascular complications and long-term liver damage [28,32]. The interaction between coffee diterpenes and dietary fibers can mitigate these adverse effects [32].

Atractyligenin, a kaurene diterpenoid, is found in coffee plants, particularly in the CS [33]. In addition, Nemzer et al. (2021) [34] identified the diterpenoid atractyligenin derivatives (carboxyatractyligenin and noncarboxyatractyligenin) in Arabica whole coffee cherry and in its two commercially available extracts, each defined by minimums of 40% chlorogenic acid and 70% caffeine, respectively. Likewise, Bichlmaier et al. (2025) [35] determined that atractyligenin and its glycosides 2-*O-ß-D*-glucosylatractyligenin, 2′-*O*-isovaleryl-2-*O-ß-D*-glucosylatractyligenin, and 3′-*O*-*β*-D-glucosylatractyligenin are significantly abundant in *Coffea arabica*, are present in roasted Arabica coffee, and are ingested with the coffee brew and converted to metabolites.

Bichlmaier et al. (2025) [35] reported that coffee-roasted powders contained 172.9–516.1 nmol/g atractyligenin and a total of 2200.1–4113.9 nmol/g atractyligenin derivatives. Consistently, the highest extraction yields and total concentrations of atractyligenin and its derivatives were observed in the French press brew (95.3%, 164.8 µM) and the Turkish brew (98.3%, 170.6 µM). Similarly, Lang et al. (2025) [30] identified atractyligenin, 2-*O-ß-*D-glucosylatractyligenin, and 3′-*O-ß*-D-glucosyl-2′-*O-*isovaryl-2-*O*-*ß*-D-glucosylatractyligenin in espresso coffee brew, with concentrations of 27.5 µM, 234.6 µM, and 30.9 µM, respectively.

Atractyligenin and its glycosides are not found in other foods and can be biomarkers for roasted coffee. In addition, Xuan et al. (2019) [33] demonstrated that noncarboxylated atractyligenin separated from CS exhibits antiphotoaging effects and may be used to treat or prevent skin photoaging.

### 2.5. Melanoidins and Eicosanoyl-5-Hydroxytryptamide

Melanoidins are high-molecular-weight brown polymers formed when amino acids and reduced sugars react via the Maillard reaction during the thermal processing of coffee beans [5,7,36]. Melanoidins are also formed during coffee brewing, contributing to their characteristic flavor, aroma, and color. The estimated amount of melanoidins per serving size for various coffee preparations (Espresso, Filter, Italian, and soluble) ranges from 99.0 to 433.0 mg. Therefore, consuming an average of four cups of coffee per day can result in approximately 1.5 g of melanoidins per day [36].

Melanoidins have been identified in various coffee by-products, such as cascara, CS, and SCG [36]. Caffeic acid and ferulic acid account for approximately 77% and 20% of the total bound phenolic compounds, respectively, and are the primary phenolic compounds integrated into coffee melanoidins. Coffee melanoidins can incorporate phenolic compounds and proteins within their polysaccharide core [37].

da Rosa et al. (2016) [38] detected 5-hydroxytryptamine (5-HT) derivatives, serotonin derivatives, in the wax layer of the coffee bean. Nemzer et al. (2021) [34] identified three 5-hydroxytryptamine derivatives (C_20_5HT, C_22_5HT, and C_24_5HT) in whole coffee cherry. Eicosanoyl-5-hydroxytryptamide (EHT), a chemical constituent found in coffee, has been shown to protect against neurodegeneration [11,34]. It also demonstrates antioxidant and anti-inflammatory effects [11]. Yan et al. (2018) [39] reported that coadministration of EHT and caffeine in mouse models reduced phosphorylated *α*-synuclein levels, preserved neuronal integrity and function, decreased neuroinflammation, and improved behavioral performance.

## 3. Advances in Coffee Bioavailability Research

Research into coffee bioavailability has moved beyond simply detecting bioactive compounds to utilize a multi-omics approach to understand how these compounds are absorbed and metabolized by the body [40,41]. Current research is also dedicated to elucidating the gastrointestinal fate of coffee bioactives and investigating novel strategies to improve their bioefficacy [42,43,44,45]. A parallel and rapidly growing trend is the appreciation of coffee by-products, such as silver skin, spent grounds, and husks, as rich, sustainable sources of functional ingredients [3]. This focus on by-products addresses two critical challenges: the impact of coffee waste and the development of novel, health-promoting food ingredients [46]. Overall, research into coffee bioavailability is increasingly focused on a more holistic understanding of its health-promoting effects. Ongoing advancements include the development of innovative delivery methods and sophisticated strategies to maximize the bioaccessibility and bioavailability of its beneficial compounds. A review of recent literature on coffee bioavailability indicates that the field is dominated by two methodological approaches: in vitro digestion models and human intervention studies. This section mainly focuses on a critical review of recent advances in human intervention studies, with an emphasis on methodological aspects.

### 3.1. Digestion Models

In vitro digestion remains the most widely used approach. Brand et al. (2024) [29] employed this approach to investigate cafestol, a lipophilic diterpene in coffee brew, demonstrating its high bioaccessibility (93.65%) and, importantly, identifying a novel carboxylic acid metabolite formed during oral digestion. Wu et al. (2023) [47] utilized an in vitro digestion model with a dialysis membrane to estimate the bioaccessibility and crude absorption rate of phenolics from fermented coffee beans, reporting that fermentation can enhance bioaccessibility. Chacón-Figueroa et al. (2025) [48] recently introduced an innovative microencapsulation technique to improve bioaccessibility. Using spray drying to encapsulate spent coffee extract within yeast cells, this technique achieved over 90% bioaccessibility in the intestinal phase. Together, these in vitro studies advance our understanding of how modifications to the food matrix can influence compound bioaccessibility, providing valuable insights into coffee bioavailability research. However, a significant methodological limitation of these studies is the lack of confirmatory human intervention trials. Without this validation, it remains unknown if the findings on coffee bean fermentation or yeast microencapsulation translate to altered pharmacokinetic profiles in actual coffee consumers. Thus, despite their analytical rigor, research findings from these studies remain preclinical indicators that require cautious interpretation.

### 3.2. Human Intervention

Human intervention studies are the gold standard for research on coffee bioavailability. They provide the most direct and reliable method for measuring the absorption, metabolism, and elimination of coffee components in the human body. Table 1 summarizes key details of 12 representative human intervention studies examining the health effects of coffee consumption. Table 2 offers a critical analysis of the most relevant methodological aspects in these studies. The studies span diverse populations (Italian, Japanese, Romanian, Spanish, Polish, US/UK, and Egyptian) and employ various designs, including randomized controlled trials, crossover designs, cohort studies, and mechanistic investigations. A general trend in these studies is a shift from basic metabolite tracking to a more targeted, high-impact clinical assessment. For example, the DECAF study [49] is the first multicenter clinical trial to assess caffeinated coffee consumption in patients with atrial fibrillation. The study enrolled 200 adults who were randomized to either consume caffeinated coffee (at least one cup daily) or abstain from all coffee and caffeine for 6 months. A key strength of this trial’s methodology is its geographic diversity, which makes its findings more applicable to a broader population. However, the trial is marked by several methodological limitations. Among these are the open-label nature of the intervention, meaning both the participants and their physicians knew which group they were in. This lack of intervention blinding can introduce various forms of bias. Another limitation is that the trial cohort consisted predominantly of older individuals (mean age: 69), which limits the generalization of the findings to younger individuals.

Complementing the DECAF trial, a large prospective cohort study investigated how adding sweeteners modifies coffee’s cardiovascular effects [50]. The study followed over 130,000 coffee consumers for a median of 12.7 years, tracking their coffee habits and cardiovascular outcomes. This large-scale study marks a significant advancement in coffee bioavailability research. Rather than simply assessing whether coffee is beneficial or harmful, it addresses the more clinically relevant question of how the choice of coffee additives influences health outcomes. A key methodological strength of this study is its assessment of specific cardiovascular disease (CVD) subtypes—coronary artery disease, stroke, heart failure, and atrial fibrillation—rather than treating CVD as a single composite outcome. This level of specificity represents a significant advance over previous work, such as Liu et al. (2022) [51], which was limited to a broader composite CVD outcome.

Overall, the major methodological advancements in current coffee bioavailability research lie in integrating genetics, sophisticated metabolomics (both targeted and untargeted), mechanistic pathways, and an understanding that coffee functions as a complex biological matrix rather than simply a source of single bioactive ingredients. However, persistent challenges remain in study design heterogeneity, the translation of findings into long-term health outcomes, and the accurate assessment of dietary intake.

**Table 1:** Key details of coffee bioavailability human trials (2021–2026).

Study	Study Category	Study Focus	Study Design	Population	Coffee Intervention/Exposure	Outcomes Measured	Key Results
[52]	Bioavailability/Pharmacokinetics	Urinary metabolomics and chemometric analysis of coffee metabolites	Human volunteer study + chemometric analysis	14 participants (8 coffee intake, 6 control), Germany	4 coffee doses over 48 h; urine collected at 8 timepoints	Urinary metabolomics (LC-MS), PCA/PLSDA, CGA metabolites	21 reported CGA metabolites identified; 5 novel bioavailable derivatives detected; endogenous hormonal metabolites emerged as key discriminators
[53]		Phenolic metabolite bioavailability and pharmacokinetics	Randomized, 3-arm cross-over	21 healthy adults (25.9 ± 0.5 y), Italy	1C: 1 cup coffee (381 μmol phenolics). 3C: 3 cups of coffee (1144 μmol) PC: 1 coffee + 2 cocoa products; 1 month	Nutrikinetics and urinary excretion of native, human phase II, and colonic metabolites	51 plasma/urinary phenolic metabolites quantified; coffee phenolics bioavailability 40–70%; metabolites average plasma concentrations < 200 nmol/L; 3C and PC induced higher circulating metabolites vs. 1C
[54]	Clinical effect	Acute coffee effects on postural control and physical function in older adults	Randomized, double-blind, crossover	22 older adults (68 ± 6 y), UK	Caffeinated coffee (3 mg/kg caffeine) vs. decaffeinated vs. placebo vs. control; 60 min post-ingestion	Postural control (COP parameters), physical function (Senior Fitness Test), perceived stability	No adverse effects on balance performance; caffeinated coffee increased sway frequency; no changes in physical function tests
[55]		Green coffee effects on miRNAs and inflammatory biomarkers in MetS	Randomized, double-blind, placebo-controlled	160 obese adults with MetS (20–60 y), Egypt	Green coffee 800 mg/day (50% chlorogenic acid), 6 months	miRNA-133a, miRNA-155, inflammatory biomarkers (resistin, TNF-α, hs-CRP, adiponectin), oxidative stress markers, blood glucose, insulin, lipid	Significant reductions in BMI, WC, BP, glucose, HbA1c, insulin, HOMA-IR, lipids; decreased miR-133a expression; reduced inflammatory markers; increased adiponectin
[56]		Roasting degree affects body composition and MetS	Randomized, controlled, single-blind crossover	38 overweight/obese adults (18–65 y), Spain	Lightly roasted coffee (LRC: 400 mg phenolics + 130 mg caffeine/cup) vs. roasted coffee (RC: 150 mg + 70 mg), 12 weeks each	Body composition (bioimpedance), MetS variables, and BP	Both coffees reduced fat mass (LRC: −1.4%; RC: −1.0%) and increased muscle mass (LRC: +0.8%; RC: +0.7%); no changes in body weight or MetS variables
[57]		Caffeine effects on exercise-induced gut damage, by genotype	Randomized, double-blind, crossover	18 adults (23.6 ± 4.1 y), UK	Caffeine 3 mg/kg vs. placebo before cycling exercise	Plasma iFABP, GI symptoms, genotype interactions	Caffeine may exacerbate gut damage; ADORA2A TT: +109% iFABP vs. 48% in C-allele carriers; no *CYP1A2* effect
[58]		Personalized nutrition (PN): genotype disclosure for caffeine reduction in slow metabolizers	Randomized controlled trial	94 *CYP1A2* C-carriers (slow metabolizers) (18–60 y), Poland	PN advice (caffeine < 100 mg/day); genotype disclosure vs. no disclosure; 20-week = + 3-year follow-up	Caffeine intake (FFQ + EMA), compliance, long-term adherence	~60% reduction in both groups; no added short-term benefit of disclosure; 3-year reduction maintained only in intervention (227.9 vs. 381.6 mg/day, *p* < 0.01); low EMA response rate (51–63%)
[59]		Coffee consumption effects on atrial fibrillation recurrence post-cardioversion	Prospective, randomized, open-label, multicenter	200 AF patients’ post-cardioversion (≥21 years old), US/Australia/Canada	Caffeinated coffee (≥1 cup/day) vs. abstinence, 6 months follow-up	AF or Flutter recurrence (primary), adverse events	Coffee consumption associated with lower AF recurrence; benefit consistent across subgroups; no significant difference in adverse events; challenges traditional view that caffeine is proarrhythmic
[50]	Epidemiological association	Unsweetened vs. sweetened coffee and CVD risk	Prospective cohort (UK Biobank)	173,614 participants, UK	Unsweetened vs. sugar-sweetened vs. artificially sweetened coffee; 12.7–year follow-up	CVD, CAD, stroke, HF, AF; PRS analysis	U-shaped association between unsweetened coffee and CVD risk; lowest CVD, stroke, and AF risk at 2–3 cups/day
[60]		Gene-coffee interactions on kidney function	Cross-sectional (J-MICC)	7468 Japanese adults (35–69 y)	Coffee intake categories: <1.5, 1.5–3, ≥3 cups/day	*eGFR*, CKD prevalence, coffee-intake-related SNPs: *AHR*, *HECTD4*, and *CYP1A2*	AHR (slow caffeine metabolizers): higher intake → 1.64 mL/min/1.73 m^2^ lower *eGFR*; *HECTD4* (frequent consumers) and *CYP1A2*: intake associated with lower CKD prevalence.
[61]		*CYP1A2* genotype interacting with B-vitamin status in MetS in	Cross-sectional observational	356 adults with MetS (18 and 75 y), Romania	*CYP1A2* rs762551 genotyping; moderate (1–2 cups/day) vs. high (≥3 cups/day) coffee intake	Folate, vitamin B12, BMI, triglycerides; gene-nutrient interactions	CYP1A2 slow metabolizers with moderate intake had higher folate and B12 vs. intermediate/rapid metabolizers; positive correlation between coffee and B-vitamins in slow metabolizers
[62]		Coffee-gut microbiome interactions and metabolomics	Multi-cohort, multi-omic cross-sectional	22,867 participants (US/UK) + 54,198 public metagenomic samples	Habitual coffee intake via FFQ; never/moderate/high intake categories	Gut microbiome composition (MetaPhlAn 4), *L. asaccharolyticus* abundance, plasma metabolomics (*n* = 438)	*L. asaccharolyticus* most strongly associated with coffee; 4.5–8-fold higher abundance in coffee drinkers; increase in blood levels of quinic acid, trigonelline, and caffeine

Abbreviations: *CYP1A2* (cytochrome P450 1A2 gene), eGFR (estimated glomerular filtration rate), EMA (ecological momentary assessment), FFQ (food frequency questionnaire), HECTD4 (HECT domain E3 ubiquitin protein ligase 4 gene), HF (heart failure), hs-CRP (high-sensitivity C-reactive protein), iFABP (intestinal fatty acid binding protein), J-MICC (Japan multi-institutional collaborative cohort), LC-MS (liquid chromatography-mass spectrometry), MetS (metabolic syndrome), miRNA (microRNA), PCA (principal component analysis), PLSDA (partial least squares discriminant analysis), PRS (polygenic risk score), TNF-α (tumor necrosis factor-alpha), GI (gastro-intestinal), BP (blood pressure).

**Table 2:** A critical review of methodological advances in coffee bioavailability human trials.

Methodological Aspect	Key Studies	Strengths/Recent Advances	Weaknesses/Challenges	Future Methodological Priorities
Genetic classification and personalized response	[57,58,60,61]	Move towards personalized nutrition: Studies are actively genotyping for key polymorphisms like *CYP1A2*, *ADORA2A*, *AHR*, and *HECTD4* to explain inter-individual variability in metabolism and response. Novel biomarker axes: Research like Popa et al. (2025) [61] explores complex interactions between genotype (*CYP1A2*) and nutritional status (B12/folate), opening new hypotheses for gene-environment interactions. The J-MICC Study (Unohara et al., 2025) [60] is a prime example of this approach in a large Japanese cohort, showing that the association between coffee intake and kidney function differs across genotypes. Behavioral intervention: Bulczak et al. (2025) [58] uniquely tests if providing genetic information leads to actual dietary change, a critical step for personalized nutrition.	Statistical power: Many studies, especially preliminary ones like Davison et al. (2025) [57], have small sample sizes for robust genotype-stratified analyses. The study itself acknowledges its “preliminary and exploratory” nature. Replication needed: Findings from specific populations (e.g., the Japanese J-MICC study, Unohara et al. (2025) [60] may not be generalizable to other ethnic groups without further validation. Mixed effectiveness: Bulczak et al. (2025) [58] showed that disclosing genetic information was no more effective than standard advice in the short term, highlighting the complexity of translating genetic knowledge into sustained behavior change.	Powering interaction studies: Ensure studies are adequately powered to detect complex gene-treatment interactions, requiring larger, multi-ethnic cohorts. Polygenic approaches: Move beyond single-gene analyses to incorporate PRS. Behavioral mechanisms: Investigate the psychological pathways (e.g., risk perception, motivation) through which genotype disclosure influences dietary adherence. Standardized genotyping panels: Adopt consensus panels of coffee-relevant polymorphisms (e.g., *CYP1A2*, *ADORA2A*, *AHR*, *HECTD4*) across studies to enable meta-analyses and replication across studies.
Advanced bioanalytical and metabolomics approaches	[52,53,59]	Comprehensive metabolite profiling: Said et al. (2023) [56] demonstrated a major advance by using both targeted and untargeted LC-MS approaches with chemometric modeling. This allows for the discovery of novel biomarkers. High-accuracy quantification: Mena et al. (2021) [52] accurately quantified 51 phenolic metabolites using numerous authentic standards. This led to higher estimates of phenolic bioavailability (40–70%). Multi-omics integration: Manghi et al. (2024) [59] combine metagenomics, plasma metabolomics, and in vitro experiments to link a specific microbe (*L. asaccharolyticus*) to coffee consumption and specific metabolites.	Clinical relevance of metabolomic findings: While Said et al. (2023) [56] excel at discovering metabolites (like hormonal changes), they do not establish the clinical bioactivity or causal role of those compounds. The authors correctly note that identified metabolites “could be used as potential biomarkers of exposure.” Observational nature of multi-omic links: The multi-cohort study by Manghi et al. (2024) [59] is largely cross-sectional and cannot prove causality, though it generates powerful hypotheses supported by well-executed in vitro work.	Link metabolites to health: Future studies should link discovered metabolites to functional outcomes using targeted mechanistic studies. Standardized analytical workflows: Develop and adopt reference standards and Consensus LC-MS/MS methods for coffee metabolite quantification to improve cross-study comparability. Longitudinal multi-omics: Integrate metagenomics, metabolomics, and clinical outcomes in longitudinal cohorts to establish temporal and causal relationships. Single-cell and spatial metabolomics: Apply emerging technologies to understand metabolite localization and cellular responses within target tissues.
Intervention design and real-world relevance	[52,54,55,62]	Robust crossover designs: Studies like Fernández-Cardero et al. (2024) [55] and Richardson et al. (2024) [62] use randomized, controlled crossover designs, which are excellent for controlling high inter-individual variability. Richardson et al. (2024) [62] also used a rigorous double-blind design with a successful blinding index. Focus on functional and mechanistic outcomes: Richardson et al. (2024) [62] investigate a meaningful functional outcome (balance in older adults), while Khedr et al. (2024) [54] explore deep mechanistic biomarkers (miRNAs). Real-world patterns: Mena et al. (2021) [52] explicitly test “different patterns of consumption” (single vs. divided doses), making findings more applicable to how people drink coffee.	Acute vs. chronic disconnect: The acute, single-dose design of Richardson et al. (2024) [62] cannot predict long-term effects. “Preliminary” nature: Fernández-Cardero et al. (2024) [55] provide “preliminary insights,” with the authors noting the “high interindividual variability” and a small sample size that limits the strength of their conclusions. Lack of dietary control: Khedr et al. (2024) [54] advised a balanced diet but did not strictly control it, introducing a potential confounder, although both groups received the same advice.	Chronic vs. acute designs: Prioritize long-term intervention studies that assess sustained effects on clinically relevant endpoints. Washout and carryover: In crossover trials, extend washout periods and analytically verify compliance (e.g., via urinary metabolites) to minimize carryover effects. Personalized dosing: Move beyond fixed doses (e.g., “3 cups/day”) to, for instance, weight-adjusted or genotype-guided dosing strategies.
Clinical and epidemiological rigor	[49,50,54,60]	Well-designed RCT: The DECAF Trial (Wong et al., 2026) [49] is a multi-center, randomized trial using an objective, clinically relevant endpoint (atrial fibrillation episodes via implantable monitor). In-depth exposure profiling: Yang et al. (2026) [50] provide significant methodological advances by distinguishing between unsweetened, sugar-sweetened, and artificially sweetened coffee. Novel mechanistic biomarkers: Khedr et al. (2024) [54] explore epigenetic mechanisms (miRNAs), moving beyond traditional inflammatory markers. Gene-environment interactions: The J-MICC Study (Unohara et al., 2025) [60] exemplifies the integration of genetic analysis into large-scale epidemiology to uncover heterogeneous effects that would be missed in a one-size-fits-all analysis.	Limitations of cohort studies: Despite sophisticated statistical adjustments, the observational nature of the large study by Yang et al. (2026) [50] allows for potential residual confounding. Likewise, the cross-sectional design used by Unohara et al. (2025) [60] in the J-MICC study hinders causal inference. Substitute biomarkers: Khedr et al. (2024) [54] use surrogate markers (miRNAs) whose direct link to long-term health outcomes is not yet fully established. Open-label design: The DECAF Trial (Wong et al., 2026) [49] is open-label, introducing potential for performance bias.	Better placebos: Develop caffeine-matched, flavor-matched placebo for blinding, reduce biases in open-label trials. Hard clinical endpoints: Prioritize RCTs with hard clinical endpoints (e.g., CVD events, mortality) over surrogate markers (miRNAs). Harmonize outcome definitions: Adopt standardized definitions for CVD subtypes and other outcomes to facilitate cross-study comparisons and meta-analyses.
Consideration of the food matrix	[52,55,62]	Matrix comparison: Fernández-Cardero et al. (2024) [55] directly compared two coffees with different roasting levels and, consequently, different phytochemical profiles (LRC: ~1200 mg phenolics/day vs. RC: ~450 mg/day). This design helps isolate the effect of the phenolic content. Whole food approach: Richardson et al. (2024) [62] use coffee as the vehicle, acknowledging that its effects may differ from individual components. Complex mixture: Mena et al. (2021) [52] investigate a mixture (coffee + cocoa product), reflecting real-world dietary complexity where foods are not consumed in isolation.	Isolating specific compounds: While comparing whole matrices is valuable, it remains difficult to attribute effects to specific components. The superiority of LRC in Fernández-Cardero et al. (2024) [55] could be due to its higher phenolic content, its different caffeine level, or a synergy between compounds. Lack of standardization: Despite these advances, there is still no universally standardized “coffee” intervention, making direct comparisons across different studies difficult.	Isolate components: Develop a “bottom-up” approach to systematically add or remove individual coffee components to isolate their independent and synergistic effects. Full matrix characterization: Prioritize comprehensive phytochemical profiling (including melanoidins and diterpenes) of any coffee intervention used in clinical trials. Explore coffee by-product bioactive components: Translate the extensive preclinical findings into human intervention studies.

Abbreviations: *CYP1A2* (cytochrome P450 1A2), *ADORA2A* (adenosine A2A receptor), *AHR* (aryl hydrocarbon receptor), *HECTD4* (HECT domain. containing 4), LC-MS (liquid chromatography-mass spectrometry), miRNAs (microRNAs), J-MICC (Japan multi-institutional collaborative cohort), DECAF (does eliminating coffee avoid fibrillation?), LRC (light roast coffee), RC (roast coffee), RCTs (randomized controlled trials).

### 3.3. Chlorogenic Acid Metabolic Map

**Figure 1:**
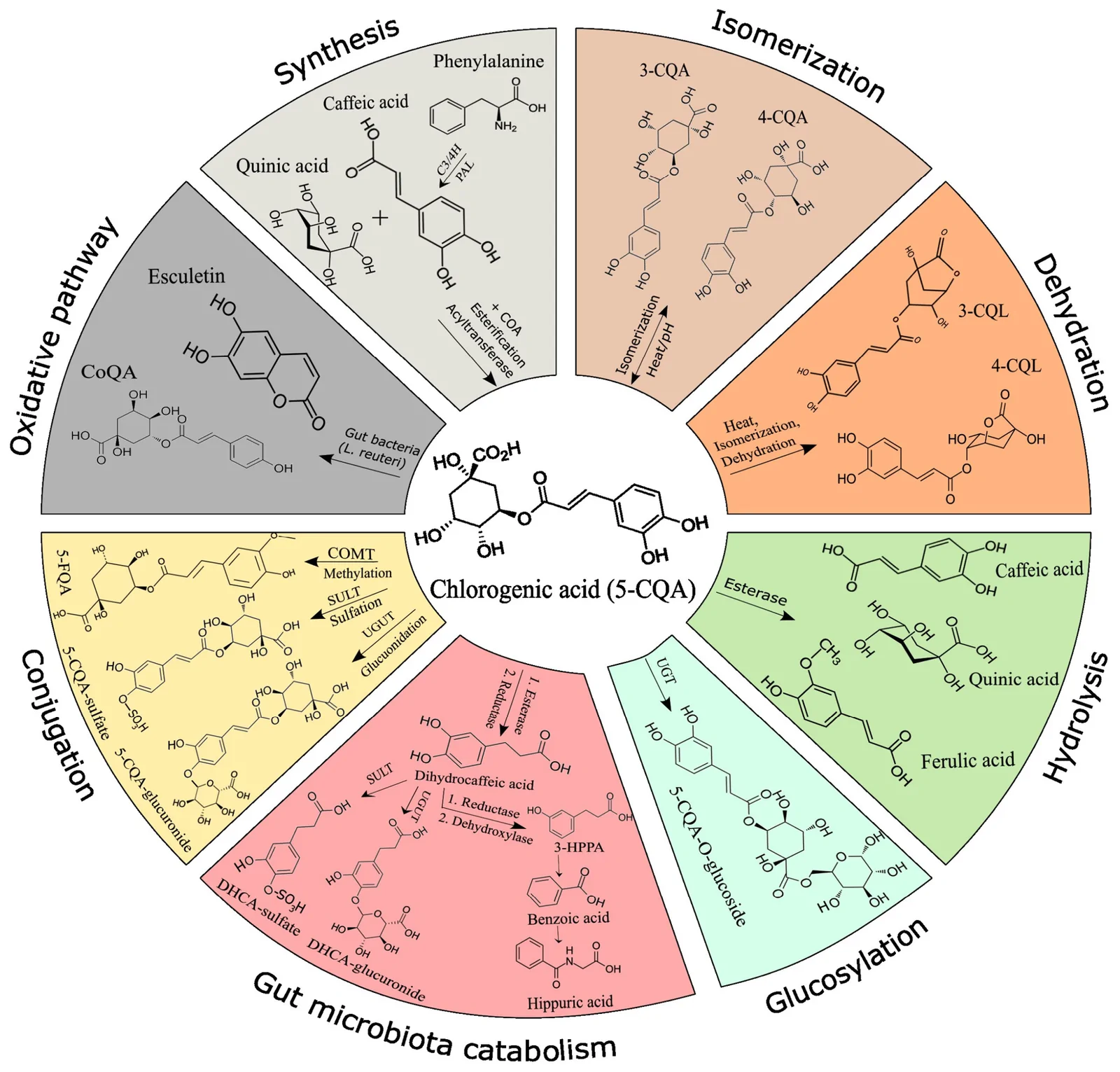
Coffee chlorogenic acid (5-CQA) metabolic map.

The health prevention potential of chlorogenic acids, particularly the predominant 5-O-caffeoylquinic acid (5-CQA) isomer, is directly linked to their complex metabolic fate. Figure 1 shows a metabolic map of chlorogenic acid (5-CQA) in coffee. This metabolic journey involves a sophisticated network spanning plant biosynthesis, food processing transformations, host phase II metabolism, and gut microbial catabolism and transformation. Biosynthesis of chlorogenic acids begins with phenylalanine, which is converted to caffeic acid via the phenylpropanoid pathway [63]. Quinic acid incorporation is catalyzed by hydroxycinnamoyl-CoA quinate hydroxycinnamoyl transferase (HQT), esterifying quinic acid with caffeoyl-CoA to produce 5-CQA, while p-coumarate 3′-hydroxylase (C3′H) introduces the catechol structure [63].

CGA undergoes several non-enzymatic transformations, particularly during food processing. During coffee roasting, thermal conditions induce isomerization of 5-CQA to 3-CQA and 4-CQA via intramolecular acyl migration [64,65], as well as dehydration producing chlorogenic acid lactones (3-CQL and 4-CQL) through water loss from the quinic acid moiety [66]. Following ingestion, hydrolysis of the caffeic acid–quinic acid ester bond occurs via microbial cinnamoyl esterases [67,68]. This hydrolysis step is critical for releasing caffeic acid, which serves as the primary substrate for subsequent metabolism. Glucosylation also represents an important modification pathway, with UDP-glucose:trans-cinnamate glucosyltransferase capable of accepting caffeate as a substrate [69]. After release from quinic acid, caffeic acid is rapidly absorbed and undergoes extensive conjugation. In the small intestinal epithelium and liver, three primary pathways operate: sulfation by sulfotransferases to caffeic acid-3′-*O*-sulfate; methylation by catechol-*O*-methyltransferase (COMT) to ferulic acid, which can be further sulfated to ferulic acid-4′-*O*-sulfate; and glucuronidation by UDP-glucuronosyltransferases to caffeic acid-*O*-glucuronide [70,71,72]. The parent compound yields 5-CQA-sulfate and 5-CQA-glucuronide as circulating metabolites, while 5-FQA represents a methylation-derived conjugate [70,73]. The fraction escaping upper GI absorption (estimated at approximately 70% of intake) reaches the colon for microbial catabolism via reductive and oxidative pathways [74].

The classical reductive pathway begins with microbial reduction of caffeic acid to dihydrocaffeic acid (DHCA) [75]. DHCA is then dehydroxylated to 3-(3-hydroxyphenyl)propionic acid (3-HPPA), which undergoes β-oxidation to benzoic acid. Benzoic acid is finally conjugated with glycine in the liver to form hippuric acid, the major urinary excretion product [76]. DHCA sulfate and DHCA glucuronide appear in circulation with a time-to-maximum concentration of 5–10 h, reflecting colonic origin [71]. The oxidative pathway involves *Lactobacillus reuteri* hydrolyzing 5-CQA to esculetin (6,7-dihydroxycoumarin), which improves intestinal epithelial barrier integrity [77]. Coumaroylquinic acid has also been identified as an intermediate in alternative microbial routes [77]. The metabolic map of chlorogenic acid thus reveals a highly integrated system in which CGA also modulates gut microbial composition by promoting beneficial *Lactobacillus* species [78], underscoring the need for continued investigation into factors that influence individual responses to chlorogenic acid-rich coffee diets.

### 3.4. Non-Extractable Polyphenols (NEPPs)

Polyphenols are a category of compounds found in plants, fruits, and vegetables. Polyphenols exhibit antioxidant, anti-obesity, anti-diabetic, anticoagulant, cardioprotective, antithrombotic, and anti-inflammatory properties [79,80]. They are classified as extractable polyphenols (EPPs) and non-extractable polyphenols (NEPPs) [79]. EPPs are partially absorbed in the small intestine because they are not bound to other food components and are therefore bioaccessible [81]. These soluble compounds are primarily degraded by colonic bacteria [79]. By contrast, NEPPs are polyphenols that are not easily extracted because they are macromolecules bound to other food components, such as fibers, proteins, or lipids, and must be unbound and bioaccessible before absorption [79,81]. NEPPs are insoluble and nondigestible and are minimally affected by fermentation microbes because they are neither released nor absorbed in the gastrointestinal tract [79]. Due to their association with dietary fiber and other components, most NEPPs reach the lower gastrointestinal tract nearly intact [79,82]. After reaching the colon, polyphenols are transformed and absorbed by the local microbiota into new small-molecule compounds [79,80,82]. Several of these metabolites have been shown to exert diverse health effects [79,80,82].

The bioaccessibility, bioavailability, and biochemical activity of polyphenols are determined by their interactions with dietary fibers present in food [79,80]. NEPPs and dietary fibers are associated with increased production of short-chain fatty acids (SCFAs), increased volume of fecal bulk, and decreased intestinal transit time [83]. Their synergistic effects enhance NEPP fermentation and elevate metabolite levels, thereby improving physiological outcomes [79,83]. In addition, the gut microbiota interacts with antioxidants and phytochemicals, including phenolics bound to dietary fibers [79,80]. Consequently, numerous NEPPs reach the small intestine, where microbial fermentation generates metabolites such as phenolic acids, SCFAs, and phenolic–SCFA conjugates [79]. Importantly, metabolites derived from NEPPs remain in the human body for longer durations than those produced from EPPs, thereby exerting prolonged biological effects. NEPPs and related phenolic metabolites can persist in human plasma for 3–4 days following ingestion, modulating low-grade inflammation and mediators of cell-signaling pathways [83].

For polyphenols to exert beneficial health effects, they must reach sufficient concentration in target tissues. Evaluating the impact of polyphenol intake on human health requires understanding their concentrations, bioaccessibility, and bioavailability throughout the gastrointestinal tract [81].

### 3.5. Proteins

Ribeiro et al. (2021) [84] reported that prior to simulated gastrointestinal (GI) digestion, the soluble protein content ranged from 51 to 64% in green, roasted, and SCGE. Following vitro-simulated GI digestion, protein solubility increased significantly to over 80% in roasted coffee and SCGE, an increase attributed to proteolysis [83].

Lu et al. (2026) [85] detected dominant protein bands at 17.0–26.0, 34.0–43.0, and 55.0–72.0 kDa, which were reduced after roasting. In contrast, high-molecular-weight bands (>180 kDa) appeared exclusively in roasted samples, suggesting substantial protein retention. Lu et al. (2026) [85] quantified peptides in roasted coffee, particularly thermostable fragments of storage proteins. These identified peptide markers indicate changes in the roast-induced proteome.

Ribeiro et al. (2021) [84] identified several bioactive peptides in the 11S storage globulin protein of *C. arabica*, with dipeptidyl peptidase-IV (DPP-IV) inhibitors and angiotensin-converting enzyme (ACE) inhibitors occurring most frequently. This study was the first to report these biological activities in coffee storage protein. The protein also contains peptide sequences with additional activities, though at lower frequencies, including DPP-III inhibitory, stimulatory, antioxidant, renin-inhibitory, regulatory, neuropeptide, anti-amnestic, immunomodulatory, calmodulin-stimulated phosphodiesterase (CaMPDE)-inhibitory, and antibacterial properties. Several 11S globulin peptide sequences demonstrate multiple biological activities. These findings indicate that the digestion of coffee 11S globulin may produce bioactive fragments that are absorbable in the intestinal tract, particularly those with DPP-IV and ACE-inhibitory activities.

## 4. Factors Influencing Coffee Bioavailability

### 4.1. Roasting and Brewing Techniques

#### 4.1.1. Green Coffee and Roasted Beans

Green coffee is a better source of chlorogenic acids (CGAs) than roasted coffee because most polyphenols degrade during roasting. Green coffee has a protective effect on the liver; however, as the degree of roasting increases, the hepatoprotective effect decreases due to the presence of CGAs in the plasma [86].

Most of the coffee consumed is roasted. Coffee roasting is crucial for developing coffee’s aroma, flavor, and color. The degree of roasting is evaluated by the color of the coffee beans, which are classified as light, medium, or dark roast. The roasting process causes numerous changes in CGAs, leading to their degradation. CGAs also interact with proteins and polysaccharides. During roasting, they are further transformed into low-molecular-weight Maillard reaction products, which evolve into melanoidins [86].

The majority of CGAs are attached to melanoidins in roasted coffee. Non-degraded CGAs do not lose their antioxidant activity when they are attached to the melanoidins. They show higher antioxidant activity and water solubility [86]. Melanoidins are about 25% of roasted coffee beans in dry matter and 1.79 mg/mL in the regular coffee brew [28].

Melanoidins are a significant source of dietary fiber and are resistant to digestion, allowing them to reach the colon intact. Phenolic compounds bound to melanoidins may be released during digestion due to stomach acidity and pancreatic enzymes present in the intestinal fluid [87]. Intact CGAs are stable and therefore difficult to absorb directly and efficiently across the small intestinal epithelium except for free form phenolic acids [28]. Consequently, the bioavailability may be greater than it would be from the concentrations of free CGAs in extracts. The low absorption of CGAs in the stomach may be attributed to their adsorption to dietary fiber in the case of green coffee and to fiber and melanoidins in roasted coffee beans. CGAs are then released because of increased intestinal pH and digestive enzyme activity [86].

The bioaccessibility of melanoidin-bound phenolics from coffee is reported to be 12% after the gastric stage and 23% after the intestinal stage. After 24 h of intestinal fermentation, bioaccessibility increases to 50%, suggesting further metabolism by the gut microbiota [87]. Melanoidins exhibit several beneficial effects in humans, including antioxidant, antimicrobial, anti-inflammatory, anticarcinogenic, antihypertensive, and prebiotic activities [36].

Grzelczyk et al. (2022) [86] revealed that CGA levels in the gastric phase after digestion decrease by more than 50% with roasted coffee extracts compared to those with green bean extracts. Similarly, in the small and large intestines, higher total CGA levels have been observed with green beans than with roasted beans. The roasting process primarily affects CGAs, whereas the caffeine concentration remains comparable between green and roasted beans throughout digestion. Thus, green coffee beans are a better source of free CGAs than roasted beans [86].

Proteases such as pepsin, trypsin, and chymotrypsin cleave polypeptide chains during GI digestion, releasing peptides and free amino acids that are more soluble and potentially bioactive. Variations in protein solubility before and after digestion are influenced by roasting and brewing processes. Roasting induces the formation of melanoidins, which bind to coffee proteins and decrease solubility. In contrast, hot-water extraction of SGC promotes protein denaturation, increases the exposure of water-binding sites, and enhances solubility [84]. Bhattarai et al. (2022) [88] reported that green coffee protein concentrate had the highest in vitro protein digestibility, whereas spent coffee protein concentrate had very low digestibility, which may be due to protein denaturation and/or the binding of polyphenols to proteins during coffee roasting and brewing [88].

#### 4.1.2. Degree of Roasting

Vamanu et al. (2020) [89] found that the degree of roasting significantly affects the concentration of bioactive compounds in coffee ground extracts, which are by-products of coffee production. Medium and dark roasts exhibited approximately a 50% reduction in bioactive compounds, cinnamic, and chlorogenic acids, compared to light roasts. However, roasting from medium to dark did not significantly (*p* < 0.05) affect the amounts of bioactive compounds, with average changes of 15%. The primary polyphenol carboxylic acids, cinnamic and chlorogenic acids, were most abundant in light-brown coffee grounds, with concentrations of 151.0 µg/mL and 135.1 µg/mL, respectively. Their levels decreased significantly (*p* < 0.05) by up to 50% in medium roast (72.6 µg/mL and 47.8 µg/mL, respectively) and dark roast (82.1 µg/mL and 56.7 µg/mL, respectively) compared to light roast [89]. Polyphenols from dark-roasted coffee bean extracts are available at higher concentrations after digestion in the small and large intestines than from coffee brew, with the highest bioavailability in the large intestine [86].

Vamanu et al. (2020) [89] investigated the bioavailability of phenolic acids across three stages of gastrointestinal digestion—oral, gastric, and intestinal—in relation to the degree of coffee roasting. The greatest reduction in bioavailability occurred during the gastric stage. In the oral stage, differences among extracts corresponded to the concentrations of phenolic compounds. The high stability of CGA minimized the impact of oral digestion. Higher roasting temperatures led to reduced yields and substrate bioavailability during intestinal digestion. All three coffee ground types retained measurable antioxidant and anti-inflammatory activities following simulated gastrointestinal digestion, although these activities declined with increasing roasting intensity. Light-roasted coffee exhibited the strongest antioxidant and anti-inflammatory effects in both in vitro and in vivo models, whereas increased roasting was associated with reduced bioactivity and lower phenolic bioavailability [89].

#### 4.1.3. Coffee Brew

Coffee brew contains polysaccharides such as galactomannans and type II arabinogalactans. Melanoidins are also present alongside these polysaccharides. Both polysaccharides and melanoidins resist digestion by human enzymes, enabling them to reach the colon intact and serve as substrates for the microbiota [24,32]. Due to these characteristics, these high-molecular-weight compounds improve coffee’s foamability and foam stability and are associated with health benefits, including cholesterol-lowering effects [32].

The coffee species, particularly Robusta due to its higher polysaccharide content, along with the degree of roasting and brewing method, collectively determine the content and structure of high-molecular-weight carbohydrates in the brew [24,32]. Variations in these carbohydrates influence both foam properties and the hypocholesterolemic effects of coffee dietary fiber [32]. Light-roasted coffees contain significantly less dietary fiber than medium- and dark-roasted varieties. Increased roasting reduces the polymerization and branching of galactomannans and arabinogalactans. Roasting also generates lower-molecular-weight polysaccharides, enhances solubility, and facilitates melanoidin formation [24,32].

Commercial coffee extracts and their polysaccharide-rich fractions, such as galactomannans and arabinogalactans, have been shown to influence in vitro cholesterol bioaccessibility by sequestering bile salts from solution [90]. Bile salts interact with coffee fibers, leading to cholesterol precipitation [32]. Coffee dietary fiber may affect cholesterol homeostasis by modulating the intestinal microbiota, which in turn influences the production and ratio of short chain fatty acids (SCFA). Fermentation of coffee fibers by gut bacteria produces SCFA. Notably, a reduction in the acetate-to-propionate ratio during this fermentation process may decrease cholesterol synthesis by altering metabolic pathways involved in cholesterol biosynthesis. Moreover, bacterial populations promoted by coffee fibers can modulate the concentrations of primary and secondary bile salts, both of which influence cholesterol homeostasis [24,90].

#### 4.1.4. Coffee By-Products

Coffee by-products contain substantial amounts of dietary fiber, including both soluble (SDF) and insoluble dietary fiber (IDF), each with distinct physiological functions. SDF helps regulate blood glucose levels and supports gastrointestinal health. IDF enhances digestive processes by increasing satiety, promoting bowel regularity, and potentially reducing the risk of certain cancers [91].

Coffee parchment, a by-product of coffee processing, is a significant source of dietary fiber, consisting almost entirely of the insoluble fraction (64.3–92.6 g/100 g). Hemicelluloses, primarily composed of xylose (28%), are the predominant polysaccharides in coffee parchment, followed by cellulose, which is mainly composed of glucose (6–12%), and pectic polysaccharides, which consist exclusively of uronic acids (4–7%). Unlike CS and SCG, the dietary fiber in coffee parchment lacks galactomannans and arabinogalactans [92].

CS, a by-product of coffee roasting, contains glucose (51–56%) and xylose (26–30%), which are the primary monomers of IDF. It also contains rhamnose (27–34%), arabinose (18–24%), mannose (17–19%), and galactose (8–14%), which are the predominant monomers in SDF [93]. Polysaccharides such as rhamnogalacturonans, type II arabinogalacturonans, galactomannans, and glucomannans present in coffee beans likely account for the observed rhamnose, arabinose, mannose, glucose, and galactose contents [93]. The total dietary fiber (TDF) content in CS from *C. arabica*, *C. canephora*, and a blend of approximately 70% *C. arabica* and 30% *C. canephora* (CS pellets) has also been reported [93]. C. arabica CS exhibited the highest TDF content (67.0 g/100 g dry weight), followed by C. canephora CS (62.0 g/100 g dry weight) and CS pellets (59.1 g/100 g dry weight). In all three CS samples, the IDF fraction (56.0 g/100 g CS C. arabica, 53.2 g/100 g CS *C. canephora*, 46.0 g/100 g CS pellets) exceeded the SDF content (11.0 g/100 g CS C. arabica, 8.8 g/100 g CS *C. canephora*, 13.1 g/100 g CS pellets). Similarly, Candela-Salvador et al. (2026) [94] reported TDF values ranging from 71.8 to 76.9 g/100 g dry weight, with a high IDF fraction (54.0–60.6 g/100 g) and SDF values ranging from 11.8 to 19.3 g/100 g in CS from *C. arabica* and *C. canephora* collected from six different countries. Dietary fibers derived from coffee by-products are classified as antioxidants due to their demonstrated antioxidant activities and are like those of fruits and fresh vegetables [95].

### 4.2. Food Matrix Interactions with Coffee—Sweeteners (Sugar and Honey), Milk and Creamers, Spices (Ginger, Cardamon, Cinnamon)

Coffee is commonly consumed with additives such as sweeteners, milk, and spices. This section describes the effects of additives on coffee bioavailability.

#### 4.2.1. Sweeteners

Nakilcioğlu-Taş (2018) [96] reported that adding sugar during brewing reduced both polyphenol content and antioxidant activity in Turkish coffee brews. Dark-roasted Turkish coffee with sugar contained the lowest levels of polyphenols and flavonoids (209.5 mg gallic acid equivalents (GAE)/100 mL and 15.5 mg GAE/100 mL, respectively) among the samples tested. Conversely, light-roasted coffee without sugar exhibited the highest polyphenol and flavonoid content (337.2 mg GAE/100 mL and 164.7 mg GAE/100 mL, respectively). Additionally, medium-roast coffee brewed without sugar had the highest antioxidant activity (66%) using 2,2-diphenyl-1-picrylhydrazyl (DPPH), whereas dark-roast coffee with sugar showed the lowest (37%). These findings suggest that consuming coffee brews without sugar yields the greatest health benefits [96]. Few studies have assessed the effects of adding sugar to coffee. Studies are needed on the bioavailability of sugar in coffee.

Honey is generally recognized for its health benefits, which include antioxidant, anticancer, antibacterial, and anti-inflammatory properties. Polyphenols are the primary phytochemicals responsible for these effects. Polyphenols in honey demonstrate greater bioavailability than those found in other functional foods. This increased bioavailability is attributed to the absence of a complex food matrix, minimal food interactions, and limited exposure to chemical reactions such as extraction or decoction, which can compromise polyphenol quality. Consequently, honey’s pharmacological and preventive actions are more effectively delivered to target tissues. These effects modulate the function of inflammatory cytokines and may decrease the severity of chronic diseases [97].

The most recent study available, conducted by Celep and Yeşılada (2017) [98], demonstrated that adding honey as a sweetener increases total phenolic and flavonoid content, as well as antioxidant capacity, in both soluble and brewed coffee. These activities are key parameters for assessing antioxidant capacity. However, Celep and Yeşılada (2017) [98] also reported significant decreases in total phenolic and flavonoid contents after in vitro digestion. There were also decreases in free radical scavenging activity and metal-reducing capacity in coffee and honey, with coffee showing the greatest decrease after digestion. The observed reductions in phenolic and flavonoid content may be attributed to pH changes during digestion, particularly in the alkaline environment of the intestines. Gastrointestinal enzymes may also contribute to this reduction by liberating phenolics from the sample matrix. However, increases in the serum-available fractions of coffee plus honey combinations, compared to those of coffee alone, were observed (*p* < 0.05). This may suggest that the phenolic compounds of honey are readily bioavailable [98]. More studies are needed on the bioavailability of honey polyphenols in coffee.

#### 4.2.2. Milk and Creamers

Milk is commonly added to coffee to enhance flavor. Interactions between milk proteins and coffee polyphenols form protein–phenolic complexes that influence both bioavailability and antioxidant properties [99,100,101]. Chlorogenic acid (CGA) and caffeic acid (CA), the primary coffee polyphenols, are chemically unstable. Adding milk increases the chemical stability of these polyphenols. Non-covalent interactions between polyphenol molecules and amino acid residues in proteins are responsible for this effect. Evidence indicates that CGA and CA exhibit a higher affinity for caseins, the main milk proteins. Caffeic acid interacts with the proline side chains of proteins via hydrophobic interactions, thereby further enhancing the chemical stability of coffee polyphenols in milk [102].

Although coffee phenolics are generally unstable in the gastrointestinal tract, their interactions with milk proteins can protect them from degradation during digestion. However, this protective effect also alters the phenolic content of coffee; adding milk reduces total phenolic content, especially total flavonoids, in both regular and decaffeinated coffee [101]. Simultaneous consumption of coffee and milk can reduce CGA absorption in humans by binding to it [100]. Consequently, increasing the amount of milk added to coffee decreases antioxidant activity and phenolic bioavailability [28]. The magnitude of this effect depends on milk protein concentration, with higher concentrations leading to greater phenol binding in coffee–milk beverages [99]. Adding milk or sugar to coffee alters the bioavailability of milk proteins, such as caseins, and diminishes the free-radical-scavenging properties of polyphenols, thereby reducing their associated health benefits [99]. Therefore, the antioxidant activity of coffee and the bioavailability of phenolic compounds decrease as milk addition increases [28].

Quan et al. (2020) [103] found that the antioxidant activity of all coffee samples with milk addition at 1:1.7 (milk: coffee *v*/*v*) decreased in vitro digestion. The bioaccessibility of phenolic compounds in coffee beverages with no milk, skim milk, and whole milk was reduced by 29.2%, 21.1%, and 28.5%, respectively, after high-pressure homogenization processing and by 14.7%, 33.8%, and 34.2%, respectively, after pasteurization. Whole milk exhibited a better protective effect on phenolic bioaccessibility than skim milk. More studies are needed to determine the optimal milk-to-coffee ratio that reduces the loss of polyphenol bioavailability when coffee is consumed with milk.

Soares et al. (2021) [104] investigated the effects of phenolic compounds from espresso capsules, focusing on caffeine and its interaction with milk during in vitro digestion. Both orally and gastric-digested samples, whether regular or decaffeinated, contained lower levels of caffeine and phenolic acids than undigested samples. The decaffeinated beverage contained the highest concentrations of all quantified phenolic acids. As a prooxidant, caffeine may promote the oxidation of certain phenolic compounds during roasting, potentially accounting for their reduced levels in regular coffee compared with decaffeinated coffee. All phenolic acids were present in the gastric phase, but only caffeic acid was bioaccessible in the intestinal phase, with a recovery rate of up to 48%. Caffeine recovery in the intestinal phase ranged from 40% to 44% in regular coffee samples. Regular coffee samples exhibited prooxidative activity during the intestinal phase, whereas decaffeinated coffee inhibited milk lipid peroxidation in milk models during both gastric and intestinal phases of in vitro digestion.

Yu et al. (2021) [101] reported that adding creamers to instant coffee significantly affected the bioaccessibility of CGAs and the antioxidant activity during in vitro digestion. Prior to digestion, instant coffee with creamer A (CRA), creamer B (CRB), and skim milk (SM) showed similar CGA bioaccessibility, approximately 63.0 mg/100 mL, which was lower than that of instant coffee alone (IC) at 67.7 mg/100 mL. This reduction suggests increased complexation of milk proteins with CGA. During gastric digestion, CGA in IC, CRA, and CRB remained stable, indicating strong stability in acidic conditions. In contrast, SM exhibited a marked decrease in CGA bioaccessibility (55.2 mg/100 mL), suggesting greater formation of protein–CGA complex during gastric digestion [101].

CGAs degrade during pancreatic digestion due to their limited stability under neutral conditions, leading to a substantial decrease in bioaccessibility. Following pancreatic digestion, the bioaccessibility of total CGAs in IC, CRA, CRB, and SM was 26%, 41%, 52%, and 84%, respectively. These findings suggest that higher protein concentrations in creamers and skim milk are associated with increased CGA bioaccessibility [101].

#### 4.2.3. Spices

The spices cinnamon, ginger, and cardamom are commonly incorporated into coffee beverages to enhance aroma, flavor, and antioxidant content, thereby offering additional health benefits to consumers [105]. Coffee and these spices exhibit several shared functional properties, including antioxidant, antidiabetic, cardioprotective, hepatoprotective, anti-inflammatory, and immunomodulatory effects [106,107].

The predominant phenolic acids in coffee extracts were caffeoylquinic acids and their isomers, whereas in cinnamon, they were cinnamic acid, proanthocyanidins, and coumarin. CGA for coffee extract and cinnamic acid for cinnamon were studied. For both coffee and cinnamon, radical scavenging activity and lipoxygenase (LOX) inhibitory activity increased after simulated gastrointestinal digestion. However, interaction between coffee phenolics and cinnamon phenols resulted in reduced radical-scavenging activity before and after digestion. LOX inhibition was induced before digestion but reduced after digestion for the mixture [105].

Coffee and ginger were studied with respect to both the interactions between individual compounds and the influence of in vitro gastrointestinal simulation. The radical-scavenging activity of coffee extracts was higher than that of ginger, and both increased with in vitro digestion. However, caffeic acid from ginger exhibited a higher antioxidant capacity than that of CGA from coffee. The coffee–ginger combination exhibited synergistic effects on radical-scavenging activity after in vitro digestion. LOX inhibitory activity was antagonistic before digestion and synergistic afterward [105].

Cardamom is another functional coffee supplement. CGA in coffee and vanillic acid, the most predominant compound in cardamom, was studied. Coffee extracts showed higher antioxidant capacity than cardamom, while cardamom extracts exhibited greater lipoxygenase (LOX) inhibitory activity. Notably, in vitro digestion increased these properties for both extracts [105]. Coffee had a high ferric-reducing antioxidant power (FRAP) value that decreased after digestion, whereas cardamom FRAP values increased. Furthermore, synergistic interactions were observed between the antiradical compounds of coffee phenolics and the LOX-inhibitory activity of cardamom phenolics, but both became antagonistic in vitro [105].

Another study by Qureshi & Patel (2023) [108] showed higher antioxidant activity in black (40%) and spice coffee (containing cloves, nutmeg, cardamom, and cinnamon) (31.5%) preparations than in lemon, mint, and milk coffee preparations. Cardamom was the only spice to show significant antioxidant activity among the four tested. The antioxidant activity further improved when coffee was prepared with water, coffee powder, cardamom, and honey. Qureshi & Patel (2023) [108] demonstrated that adding cardamom and honey to black coffee increased radical scavenging activity by 21% to 61%. In contrast, replacing water with milk and honey with stevia leaves reduced radical-scavenging activity by 8% to 52%. However, this was still higher than the antioxidant potential of black coffee and may be considered an antioxidant-rich beverage for consumers. Therefore, coffee prepared with or without milk and containing cardamom and/or honey or stevia leaves provides a suitable source of antioxidants associated with plant polyphenols and coffee components.

## 5. Strategies to Improve Coffee Bioavailability

### 5.1. Encapsulation

Encapsulation safeguards core materials from environmental factors, improves their stability and bioavailability, and enables controlled release [109]. Encapsulation is an emerging technique that applies a thin film to the surface of core materials. These core materials, such as coffee polyphenols, are protected from adverse environmental conditions through this process [110]. The encapsulation process involves two primary material types. The first consists of bioactive compounds or extracts, also known as pure materials, core materials, internal phases, fillers, or actives. The second type comprises packaging materials, including gums, proteins, lipids, natural polymers, modified polymers, and synthetic polymers. These are also referred to as wall materials, coating materials, capsules, membranes, or carriers [111].

Spray-drying and freeze-drying are the most common drying methods used in many applications to encapsulate coffee or coffee by-products and produce powder. Araújo et al. (2022) [112] reported high levels of encapsulated phenolic compounds and antioxidants in dried SCG extracts prepared from a combination of wall materials (albumin, maltodextrin, and gum Arabic) obtained by spray- and freeze-drying techniques. Results of these drying techniques did not differ significantly (*p* > 0.05). Albumin showed the worst performance in retaining bioactive compounds, whereas gum Arabic combined with maltodextrin performed better at preserving them.

Spray-drying generally provides high encapsulation efficiency and high chemical stability against harsh conditions. However, exposure to hot-air drying can degrade sensitive bioactive compounds, limiting the applicability of spray-drying [113,114]. In contrast, freeze-drying is primarily used to encapsulate thermosensitive compounds. This method is limited by prolonged processing times (24–48 h) and the requirement for reduced-pressure conditions, which increase energy consumption. Despite these constraints, freeze-drying remains widely used for encapsulating therapeutic compounds, as it produces high-value products that require bioactive compounds with enhanced biological activity [113].

### 5.2. Polyphenols, Their Challenges, and Nano-Encapsulation

Polyphenols exhibit a broad range of biochemical activities, including antioxidant, anti-inflammatory, anti-tumor, anti-diabetic, neuroprotective, and cardioprotective effects [115,116,117]. Despite these benefits, polyphenols face significant limitations, including sensitivity to heat and light, low aqueous solubility, rapid metabolism, and high molecular weight, which collectively diminish their therapeutic efficacy. Furthermore, polyphenols may degrade in aqueous environments or undergo oxidation, resulting in reduced activity. Their bitter taste can also limit their application in food products and oral pharmaceuticals [115,117].

Most polyphenolic compounds exhibit poor gastrointestinal absorption, instability at physiological pH, and extensive first-pass metabolism in the liver and intestines. These factors result in low bioaccessibility, limited bioavailability, and reduced bioactivity, leading to insufficient concentrations in target tissues and diminished therapeutic potential against human diseases [115,116]. Additionally, polyphenols exhibit limited permeability across biological barriers, such as the blood–brain barrier, thereby significantly limiting their effectiveness in treating neurological disorders and diseases affecting protected compartments [115].

To overcome these limitations, nanotechnology-based delivery platforms, such as phytosomes, liposomes, micelles, solid lipid nanoparticles, nanoemulsions, self-microemulsifying drug-delivery systems, cyclodextrin complexes, dendrimers, and nanocrystals, have been utilized to optimize polyphenol administration. These systems increase solubility, permeability, and metabolic stability, thereby enabling controlled and targeted release of polyphenols, which are especially valuable in chronic inflammatory and metabolic disorders that require sustained polyphenol delivery [115]. Some of these delivery platforms have been applied to polyphenols in coffee and coffee by-products.

### 5.3. Applications of Novel Compound Delivery Systems for Coffee and Coffee By-Products as Functional Ingredients

CCE exhibits in vitro anti-aging properties and slows human skin aging [118]. Saewan et al. (2023) [119] identified chlorogenic acid (131.9 µg/mL) as the major compound in CCE. Liposomal encapsulation enhances the solubility of polyphenols, protects them from harsh gastrointestinal conditions and enzymatic degradation, and facilitates their passage across cellular membranes, including the intestinal lining and the blood–brain barrier, thereby enabling their use in the treatment of neurological and systemic diseases [115]. The liposomal encapsulation system of CCE also enhances the stability of chlorogenic acid, particularly under high-temperature conditions, and provides a more consistent, higher, and prolonged release of phenolics than free CCE. Accordingly, liposomal encapsulated CCE showed lower toxicity and slightly greater anti-aging effects on human dermal fibroblasts than CCE alone. The findings suggest that encapsulation in a lipid nanocarrier, which may enhance stability, reduce toxicity, and preserve biological activity, has potential as an active ingredient for anti-aging and skin-brightening applications in cosmetic and cosmeceutical products [119] (Table 3).

Indiarto et al. (2022) [120] developed a cocoa drink formulation containing encapsulated green coffee bean extract at concentrations ranging from 0% to 10%. Cocoa powder contains antioxidants, but its antioxidant activity may be reduced during processing. Therefore, adding green coffee extract, a powerful antioxidant, is crucial. The extract needs to be encapsulated to maintain its stability and mask its bitter taste. The results showed that increasing the encapsulated extract percentage increased polyphenol and flavonoid levels and antioxidant activity (Table 3).

Coffee husk extracts (CHE) are rich in polyphenols and are increasingly recognized as functional ingredients in food products and for diabetes management [121]. de Oliveira et al. (2024) [122] demonstrated the encapsulation of CHE with polyvinylpyrrolidone (PVP), a nonionic, biodegradable synthetic polymer that can encapsulate both lipophilic and hydrophilic compounds [123]. This property makes PVP suitable for drug delivery systems that utilize CHE, which contain both hydrophilic and lipophilic constituents [122]. Encapsulation with PVP can mask undesirable flavors, enhance the aqueous solubility of bioactive compounds, and improve their biological activity. The study identified alkaloids, phenolic compounds, monosaccharides, and sugar alcohols in both the CHE and the encapsulated product. Quercetin and caffeic acid were the predominant phenolic compounds, while trigonelline was the most abundant alkaloid, followed by caffeine. Levels of caffeic acid and theobromine remained unchanged between the two forms. The amount of total phenolic content and antioxidant activity was greater in encapsulated coffee husk extract (ECHE) than in the non-encapsulated extract [122]. Table 3 shows the effect of the ECHE on the bioactive compounds.

CS contains significant quantities of bioactive compounds, primarily chlorogenic acid and caffeine. It has been used as a functional ingredient due to its antioxidant properties, which enhance product bioactivity and inhibit lipid oxidation. However, CS extract demonstrates high water activity, which accelerates the degradation of these bioactive compounds [124]. Table 3 presents the concentrations of bioactive compounds when encapsulated with various carrier materials in CS. The study did not report the initial chlorogenic acid and caffeine contents prior to spray drying and encapsulation with carrier materials, which prevents direct comparison to assess any increase resulting from encapsulation. Iriondo-DeHond (2019) [25] reported that CS extracts contain 9.4 mg/g of 5-caffeoylquinic acid (5-CQA) in Arabica and 23.3 mg/g in Robusta, with a caffeine-to-CQA concentration ratio of 2.5 in both species.

Fathi et al. (2022) [125] encapsulated caffeine and chlorogenic acid from CS into nano-phytosomes or cholesterol-incorporated nano-phytosomes as primary coating layers using spray-drying to enhance phytochemical stability, absorption, bioavailability, and functionality (Table 3). Both types of lipid-based layers were subsequently coated with various natural polymers as secondary coating layers. Additionally, several natural polymers were assessed as primary coating layers for the silverskin-enriched extract. Natural-based carriers enable safe and targeted delivery, while polymer coatings improve stability during storage and handling and allow for controlled release. Fathi et al. (2022) [125] reported that, irrespective of coating type, the final products demonstrated high efficiency at lower dosages, supporting their suitability for oral delivery. CS products enriched with caffeine and phenolic compounds may be considered for use in dietary supplements to enhance mental and physical performance. However, the study did not address the impact of encapsulation on the concentrations of caffeine and chlorogenic acid in CS.

SCG is a by-product of extracting soluble coffee and preparing ground-bean infusions, accounting for more than 50% of the total weight of roasted coffee beans [126]. SCG is rich in bioactive compounds, particularly caffeine, chlorogenic acid, and caffeic acid [127]. These compounds are susceptible to degradation during digestive processes [128]. Ballesteros et al. (2017) [128] found that freeze-drying encapsulation with a maltodextrin coating most effectively preserved antioxidant phenolic compounds and flavonoids extracted from SCG, compared to spray-drying encapsulation, gum arabica, or a maltodextrin-gum arabica mixture coating (Table 3).

Several nanocarrier-based polyphenol-delivery systems, including liposomes, phytosomes, polymeric micelles, and self-emulsifying drug-delivery systems, are commercially available. These advances significantly enhance solubility, bioavailability, and therapeutic targeting of polyphenols, making them promising formulation strategies [115]. These are applicable to polyphenols in coffee and coffee by-products.

**Table 3:** Effects of encapsulation of coffee and coffee by-products as functional ingredients.

Coffee/Coffee By-Product	Carrier Material	Encapsulation/Drying Technology	Delivery Systems: Results/Advantages	References
Coffee cherry extract (CCE)	Liposomes (15:1, phosphotidylcholine and cholesterol)	High-Pressure Microfluidizer	After 12 weeks, the CCE encapsulation retained phenolic contents of 95, 96, and 90%, while the phenolic contents of CCE decreased to 94, 88, and 74% at 4 °C, room temperature, and 45 °C, respectively. The release rate of CCE encapsulation in the liposomes increased gradually and consistently over time, while the rate of release from free CCE increased for 24 h and then decreased from 48 h to 100 h. The liposomes demonstrated lower cytotoxicity than CCE, and they exhibited higher anti-aging effects than CCE, including NO inhibition, increased SOD activity, and anti-collagenase activities.	[119]
Green coffee bean extract (GCBE)	Maltodextrin (80%) and gum Arabica (20%)	Spray-drying	Maintains stability Masks the bitter taste Encapsulated GCBE contained a total phenolic content (TPC) and total flavonoid content (TFC) of 76.6 mg GAE/g and 22.7 mg GAE/g sample, respectively; after 0–10% of encapsulated GCBE was added to cocoa drinks, TPC ranged from 9.5 to 16.7 mg GAE/g, and TFC ranged from 3.1 to 8.0 mg GAE/g, which were significantly higher (*p* < 0.05) than those of the cocoa drink without the encapsulated GCBE. The antioxidant activity of the encapsulated green coffee bean extract was 73.2 ppm, which is considered a strong antioxidant. When 10% of the encapsulated GCBE was added to cocoa drinks, the antioxidant activity increased significantly (*p* < 0.05) to 95.4 ppm, indicating a strong antioxidant.	[120]
Coffee husk extract (CHE)	Polyvinylpyrrolidone	Circulating oven at 60 °C	Masks undesirable flavors Increases stability of bioactive compounds, improves biological activity Encapsulated coffee husk (ECH) content had greater concentrations of the following: 12% greater trigonelline, 180% greater quercetin, 17% greater caffeine Phenols bioaccessibility 71% vs. 57% in non-encapsulated CHE after simulated gastrointestinal digestion ECH demonstrated higher antioxidant activity than CHE.	[122]
Coffee silverskin (CS)	Maltodextrin, Modified starch, Arabica gum	Spray-drying	The combination of modified starch and arabica gum yielded the highest chlorogenic acid concentration of 71.8 mg/100 g, while maltodextrin resulted in the lowest concentration of 2.2 mg/100 g. The modified starch and arabica gum mixture produced the highest caffeine concentration of 309.6 mg/100 g, whereas maltodextrin produced the lowest concentration of 65.1 mg/100 g.	[124]
Coffee silverskin (CS)	Nano-phytosomes, cholesterol-incorporated nano-phytosomes	Spray-drying	Improve the protection, solubility, stability of caffeine and chlorogenic acid, absorption, bioavailability, and functionality of the phytochemicals	[125]
Spent Coffee Grounds (SCG)	Maltodextrin	Freeze-drying	Preserved antioxidant phenolic compounds (62%) and flavonoids (73%), respectively Preserved 73–86% of antioxidant activity	[128]
Spent Coffee Grounds (SCG)	*Saccharomyces cerevisiae* yeast cells	Spray-drying	Beers without additives contained 353.7 mg GAE/L total phenol concentrations Beers with the addition of unencapsulated spent coffee extract contained 747.5 mg GAE/L). Among beer samples with non-plasmolyzed microcapsules, a greater increase was demonstrated in total phenol concentration (564.9 mg GAE/L) than in beers with plasmolyzed microcapsules (463.9 mg GAE/L). Beers formulated with SCG microcapsules exhibited increased bioaccessibility of bioactive compounds in the intestinal phase, reaching 108% for plasmolyzed microcapsules and 97% for non-plasmolyzed microcapsules, compared to 39% in non-additive beer.	[48]

### 5.4. Other Applications of Novel Coffee Bioactive Compounds Delivery Systems

Otalálora et al. (2024) [129] encapsulated pink guava pulp using coffee mucilage as a wall material through freeze-drying. The study also compared maltodextrin and a 1:1 maltodextrin-to-coffee mucilage powder mixture as alternative wall materials. Pink guava is recognized as a rich source of natural carotenoid pigments and exhibits antioxidant properties. Retention of total carotenoids was higher in freeze-dried coffee mucilage microcapsules (80%) compared to maltodextrin (61%) and the combined maltodextrin and coffee mucilage (61%). Coffee mucilage contains polysaccharides, proteins, and antioxidants. The total phenol content in coffee mucilage was 3.6 mg GAE/g. Otalálora et al. (2024) [129] did not report the total phenol content of pink guava fruit. The total phenol content was greater in freeze-dried coffee mucilage microcapsules (30.2 mg GAE/g dry weight) than in maltodextrin (20.0 mg GAE/g dry weight) and the combination of coffee mucilage and maltodextrin (25.3 mg GAE/g dry weight) capsules. This increased retention is attributed to the protective effect of coffee mucilage, which serves as both a barrier and an inherent antioxidant. The findings indicate the potential of powdered coffee mucilage as a sustainable and effective wall material for encapsulation. Further research is required to confirm the bioavailability of bioactive compounds in encapsulated products.

Intranasal (IN) bioactive compound delivery systems offer a direct and efficient method for delivering therapeutics to the brain, avoiding the blood-brain barrier and enabling rapid targeting for the treatment of cancer, neurodegenerative diseases, stroke, and other conditions [130]. These systems require high encapsulation efficiency, protection of drugs from degradation or denaturation, enhanced retention within the nasal mucosa, and facilitation of drug transport across the nasal epithelium to reach brain tissue. Understanding the pathways and biological barriers encountered by intranasally administered drugs is essential for designing biomaterial-based carriers that effectively deliver therapeutics to the brain. Multifunctional hydrogel formulations encapsulating drug-loaded nanocarriers have further improved brain delivery via the IN route. IN drug delivery systems targeting brain tissues have demonstrated potential for effective treatment of neurodegenerative diseases and brain cancers [130]. This approach represents a promising strategy for enhancing the bioavailability of compounds, such as those found in coffee.

Chacón-Figueroa et al. (2025) [48] used spray drying and microencapsulation with non-plasmolyzed (NPCs) and plasmolyzed (PCs) *Saccharomyces cerevisiae* yeast cells to stabilize bioactive compounds in SCG during in vitro digestion, thereby enhancing their release throughout the gastrointestinal tract, particularly during the intestinal phase. The main compounds identified in SCG included caffeine (2.9 mg/g), chlorogenic acid (4.3 mg/g), and caffeic acid (0.9 mg/g). Capsules with plasmolyzed yeast cells contained a higher concentration of total phenols (278.4 mg GAE/g dry weight) than those with non-plasmolyzed yeast cells (192.8 mg GAE/g dry weight). The study also evaluated the use of these yeast cells in craft beer production (Table 3). The results demonstrate that microencapsulating spent coffee extract within yeast cells stabilizes and protects bioactive compounds against environmental degradation [48]. These microcapsules may serve as functional additives in the food industry.

While all delivery systems were experimentally validated in food matrix applications, using different techniques, such as physicochemical and biochemical in vitro digestion models, the liposome-encapsulated coffee CCE was uniquely validated through a human intervention study (Table 3).

## 6. Coffee’s Health Effects on Aging

Aging is inevitable, and with it comes an increase in chronic diseases, which affects the quality of life of the elderly. Coffee consumption promotes healthy aging by potentially extending longevity through its neuroprotective effects, cardiovascular benefits, metabolic support, and skin health. Coffee has been shown to be associated with reduced risks of these conditions. The composition of its primary bioactive compounds, caffeine and chlorogenic acids, contributes to these health benefits.

### 6.1. Coffee’s Neuroprotective Effect

Coffee consumption correlates with a decreased risk of several chronic diseases associated with inflammatory processes, including neurodegenerative conditions, with caffeine identified as a protective agent. Caffeine has strong antioxidant and anti-inflammatory effects against many neurodegenerative diseases, including Alzheimer’s and Parkinson’s [131].

After oral administration, caffeine is rapidly absorbed in the small intestine, with plasma concentrations peaking within 30 to 60 min. Caffeine quickly distributes throughout body tissues, including the nervous system [10,132]. Since caffeine is both water- and fat-soluble, it can cross the blood–brain barrier and exerts an antagonistic effect on adenosine receptors in dopamine-rich regions of the brain, thereby increasing dopamine release and enhancing neurotransmission. This association suggests that coffee intake may slow the progression of dementia or lower the risk of Parkinson’s disease (PD) [11].

The most prevalent polyphenol in coffee, chlorogenic acid, also has neuroprotective properties. It exhibits antioxidant and anti-inflammatory properties by inhibiting cytokine gene expression and modulating nuclear factor-kappaB activation, a signaling pathway associated with neuronal regeneration, thereby contributing to neuroplasticity [11].

Brain-derived neurotrophic factor (BDNF) is a multifunctional neurotrophin that plays a central role in neuronal development, survival, and synaptic plasticity. These processes are fundamental to learning, memory formation, and working memory [133]. Circulating BDNF has also been associated with healthy cognitive aging, and higher serum BDNF concentrations are linked to reduced risk of age-associated cognitive diseases such as AD and dementia [134].

Oral supplementation with chlorogenic acid-rich extracts from whole coffee cherry has been reported to increase the circulation of BDNF in healthy adults [135,136]. Building on these findings, Robinson et al. (2021) [137] later evaluated the neurophysiological effects of whole coffee cherry extract (WCCE) in older adults with subjective cognitive impairment. Using blood oxygen level-dependent (BOLD) functional magnetic resonance imaging (fMRI) during tasks assessing working memory and response inhibition, the authors demonstrated that a single oral dose of WCCE increased functional connectivity between the anterior cingulate cortex and brain networks involved in sensory processing and decision-making. Simultaneous, acute increases in circulating exosomal BDNF were also associated with improvements in cognitive performance, including measurements of reaction time and accuracy.

Moderate intake of three-to-five cups of coffee, equivalent to approximately 300 mg of caffeine, may reduce the overall risk of cognitive impairment by preventing or delaying the onset of progressive nervous system disorders such as PD and Alzheimer’s disease (AD) [11]. A comprehensive systematic review reported positive effects of chlorogenic acid and coffee consumption on memory, attention, executive function, alertness, and motor activity, as well as a lower risk of cognitive impairment, dementia, and AD following chronic coffee intake [138]. The widely consumed *C. canephora* and its bioactive compounds, including caffeine and chlorogenic acid, have potential neuroprotective effects against neurodegenerative diseases [139].

Lin et al. (2026) [140] explored the individual or interactive associations between coffee intake and neurodegenerative diseases among older adults. The study included 134,425 participants from the UK bank. All participants were aged 60 or older, white, and free of neurodegenerative disease. A total of 6483 individuals developed neurodegenerative diseases during a follow-up of 13.5 years. Participants were grouped by their daily coffee preference: ground, instant, and decaffeinated. Lin et al. (2026) [140] show that an increased risk of all-cause neurodegenerative diseases is associated with a daily consumption of more than four cups of coffee. The risk of vascular neurodegenerative diseases was greater with more than six cups of coffee per day. Lin et al. (2026) [140] determined that drinking up to five cups of coffee per day was associated with a risk of morbidity for primary neurodegenerative diseases and all-cause degenerative diseases. Coffee type also modulated the association between coffee intake and neurodegenerative diseases. Participants consuming 0.5–5 cups of ground coffee per day had a lower risk of all-cause neurodegenerative disease than those who never consumed coffee. Lin et al. (2026) [140] also observed that consumption of decaffeinated coffee (0.5–5 cups per day) was associated with a higher risk of primary neurodegenerative disease morbidity than that of those who never drank coffee. There was no association between *CYP1A2*, a gene regulating coffee metabolism, and coffee intake or neurodegenerative disorders.

Dong et al. (2020) [141] examined the relationships between coffee, decaffeinated coffee, caffeinated coffee, and caffeine from coffee, and cognitive performance among 2513 Americans aged 60 years or older (1214 men and 1299 women) who participated in the 2011–2014 National Health and Nutrition Examination Survey (NHANES). The findings suggest that coffee, caffeinated coffee, and caffeine from coffee are associated with improved cognitive performance, but decaffeinated coffee was not [141]. On the other hand, Shirai et al. (2019) [142] investigated the relationship between coffee consumption and cognitive decline in older adults aged 60 to 85 years using data from the National Institute for Longevity Sciences, Longitudinal Study of Aging in Japan. A Mini-Mental State Examination was used to assess cognitive function over a period of up to 6 years. The findings indicated that coffee intake did not lower the likelihood of cognitive decline in this population.

Robinson et al. (2019) [143] examined the effects of CCE consumption on cognitive performance and reported that adults with mild cognitive decline experienced significant reductions in reaction time (*p* < 0.05) for approximately 1 month. These findings indicate that taking CCE in the morning or twice daily is associated with enhanced attention span and focus. In a follow-up study, Robinson et al. (2024) [144] reported acute improvements following ingestion of 200 mg of a CCE in measures of accuracy during working memory and inhibitory control tasks in generally healthy adults aged 40–65. Moreover, a population-based study involving 145 community-dwelling older adults aged 69 to 86 years found a positive correlation between coffee consumption and cognitive performance. Over a 3-year period, two neuropsychological assessments indicated that moderate coffee consumption (up to two cups) is associated with better white matter preservation and improved cerebral blood flow in cognitively stable elders without significant cerebrovascular pathologies [145].

### 6.2. Cardiovascular Health and Coffee Metabolic Support

An observational study by Wang et al. (2025) [146], using data from 40,725 participants of the 1999–2018 National Health and Nutrition Examination Survey (NHANES), evaluated the association between coffee-drinking timing and mortality risk. The study reported that morning coffee intake may be associated with lower risks of all-cause and cardiovascular disease-specific mortality, regardless of the quantity consumed. In contrast, all-day coffee consumption was not associated with mortality among U.S. adults who did not drink coffee. Together, these findings indicate that morning coffee consumption may confer benefits for cardiovascular health. Likewise, Fan et al. (2023) [147] investigated the association between caffeine levels and stroke incidence using data obtained from 5339 individuals participating in the NHANES conducted between 2009 and 2014. Higher urinary caffeine and paraxanthine levels were correlated with a lower stroke incidence in Mexican American participants. However, no other studies have shown an association in other diverse populations.

A study of 200 patients (average age 69 years) from five hospitals in the U.S., Canada, and Australia with persistent atrial fibrillation (AF) or atrial flutter was divided into two groups and assessed for caffeinated coffee consumption. Patients in the consumption group drank at least one cup of caffeinated coffee, while patients in the abstinence group were encouraged to abstain completely from caffeinated and decaffeinated coffee and other caffeine-containing products. Results showed that caffeine intake, compared with abstinence from coffee and caffeine, was associated with a significantly lower risk of recurrence of atrial fibrillation or atrial flutter [49].

Stevens et al. (2021) [148] showed that elevated coffee consumption was associated with a minimal risk of developing heart failure later in life across three epidemiological studies: the Framingham Heart Study (FHS), the Cardiovascular Heart Study (CHS), and the Atherosclerosis Risk in Communities (ARIC) study. In a UK Biobank study, 449,563 participants (average age 58 years) were followed for more than 10 years to assess the associations between coffee subtypes and cardiovascular disease (CVD), including coronary heart disease, cardiac failure, and ischaemic stroke. Ground and instant coffee consumption were significantly associated with a reduction in arrhythmia (*p* < 0.0001) at 1–5 cups per day, but not with decaffeinated coffee. The lowest risk was observed for those who consumed four to five cups of ground coffee and two to three cups of instant coffee daily. All coffee subtypes (decaffeinated, ground, and instant) were associated with a reduced risk of CVD [149]. In a study of 131,247 individuals (average 55.7 years), most consumed unsweetened coffee (55.6%), followed by sugar-sweetened (14.1%) and artificially sweetened (5.9%). Those who consumed more than one cup of unsweetened coffee per day had a lower risk of CVD than non-consumers. However, no noteworthy cardiovascular benefit was observed with coffee containing added sugars or artificial sweeteners [50]. Overall evidence suggests that moderate coffee consumption (up to five cups daily) is associated with a reduced risk of CVD. Increased consumption may be associated with a higher risk of heart failure.

### 6.3. Skin Health

Human skin experiences both intrinsic and extrinsic aging processes. Intrinsic aging is characterized by dryness, reduced elasticity, and fine wrinkles, primarily due to decreased collagen production and a less distinct dermal–epidermal junction. In contrast, extrinsic aging is driven by environmental factors, particularly ultraviolet exposure, which accelerates the degradation of elastin and collagen, resulting in wrinkles, rough skin texture, and pigmentation alterations [150].

Coffee-derived materials, including those obtained from coffee beans and by-products such as leaves, SCG, and CS, have demonstrated anti-aging activities in vitro, in animal models, and in limited human trials. Evidence indicates that these materials can improve skin hydration, enhance barrier function, facilitate tissue repair, and mitigate damage caused by oxidative and environmental stressors. Additional research is necessary to advance the development of coffee-derived bioactives as ingredients in cosmetic and therapeutic formulations targeting skin aging [150].

Coffee cherry fruit demonstrates significant anti-aging properties, primarily due to its main constituents, caffeine, and chlorogenic acid. CCE contains a high concentration of phenolic compounds, exhibits strong antioxidant activity, and effectively inhibits collagenase. The superoxide dismutase (SOD) activity and nitric oxide inhibition of CCE are greater than those of caffeine but lower than those of chlorogenic acid. Furthermore, CCE exhibits greater 5α-reductase inhibition than chlorogenic acid, which itself is more effective than caffeine in this regard [118]. Topical application of 1% CCE cream and 0.1% CCE cleanser twice daily has been shown to improve skin appearance, minimize fine lines and wrinkles, and reduce roughness, dryness, and pigmentation. In addition, CCE reduces levels of collagenase (MMP-1) and IL-1, both of which are inflammatory markers in biopsies while simultaneously increasing collagen levels [119].

Recent upcycling initiatives have focused on reusing coffee by-products to reduce waste in the cosmetics industry. Spent coffee by-products demonstrate significant potential for use in cosmetic formulation. Ribeiro et al. (2018) [151] incorporated SCG extracts into hydrogels intended for topical application. The antioxidant capacity of SCG, combined with its inhibitory effects on elastase and tyrosinase, suggests suitability for anti-aging and skin-lightening products. Delgado-Arias et al. (2019) [152] developed an exfoliating body cream containing SCG, which exhibited both exfoliating and antioxidant properties. The formulation with 6% SCG exhibited high antioxidant and polyphenol content, as well as favorable textural attributes, such as adhesiveness and cohesiveness. Furthermore, the cream enhanced skin wettability and softness.

CS has antioxidant capacity attributed to polyphenols and other bioactive compounds, which support anti-aging properties and have great potential to improve skin health. Preventing ultraviolet damage is an effective way to reduce the effects of photoaging, a major contributor to facial wrinkles. CS extract can be used as a topical component in skin care products and as a preventive against accelerated skin aging induced by oxidative stress from chemical or physical agents (photoaging) [153].

Caffeine and bioactive compounds derived from coffee and their by-products serve as effective ingredients in the cosmetic industry. Bioactives extracted from CS and SCG enhance skin appearance by providing anti-aging, antioxidant, anti-inflammatory, antimicrobial, anti-cellulite, anti-hair-loss, and ultraviolet protection effects. Caffeine demonstrates strong antioxidant activity and is commonly incorporated into anti-aging and anti-cellulite moisturizing creams, as well as treatments for various skin disorders, including androgenetic alopecia. Research indicates that caffeine inhibits skin-aging enzymes, such as collagenase, elastase, and tyrosinase, thereby exerting anti-aging effects. However, caffeine alone cannot penetrate deeper dermal layers. Nanoscale delivery systems enhance caffeine’s dermal penetration and efficacy. This strategy offers potential for developing new caffeine-based products for skincare formulations [154].

**Figure 2:**
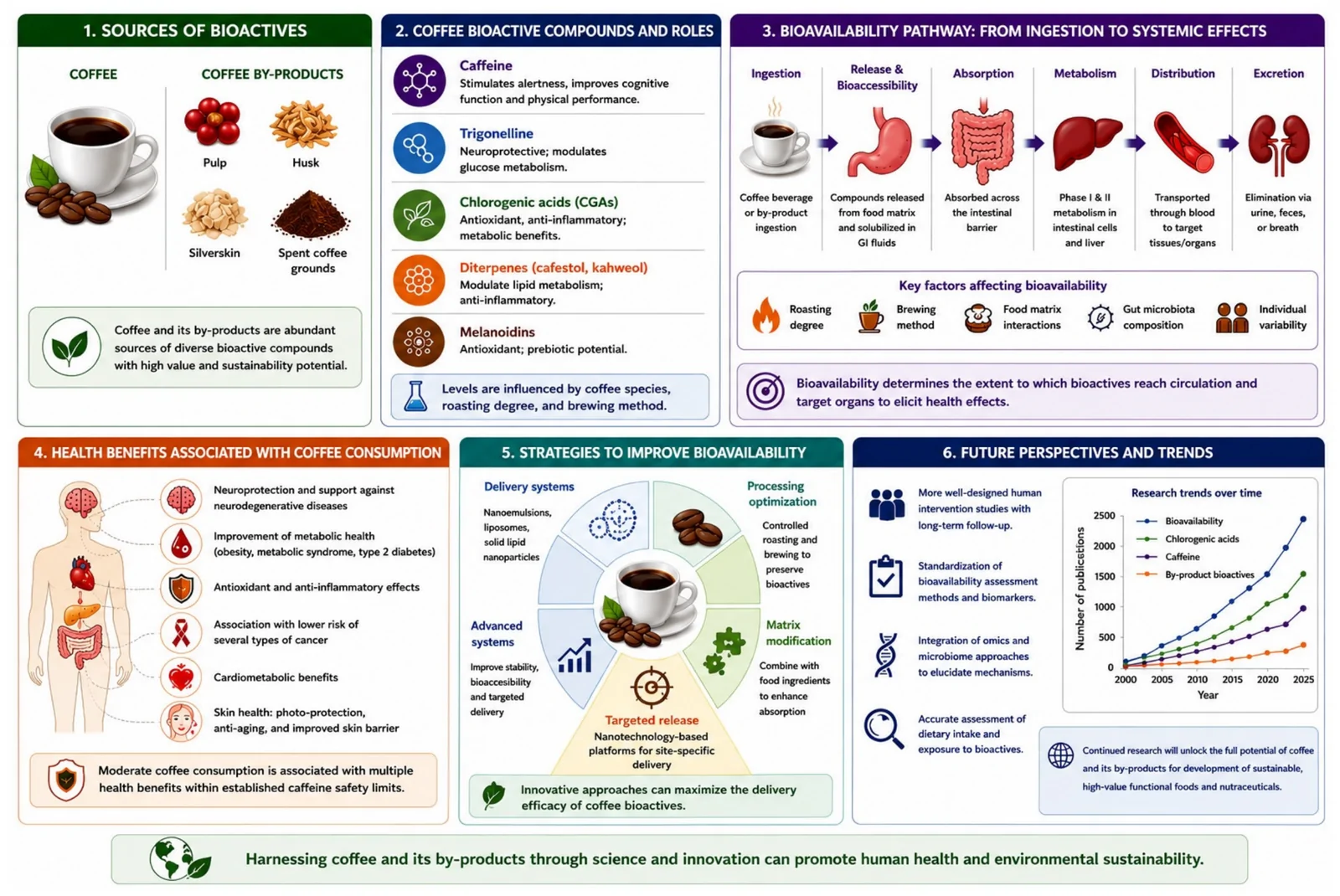
Integrated overview of bioactive compounds from coffee and coffee by-products, highlighting their major chemical constituents, gastrointestinal bioavailability pathway, factors influencing absorption and metabolism, associated health benefits, emerging strategies to enhance bioavailability, and key priorities for future research and sustainable utilization.

In summary, Figure 2 integrates the information presented in this review.

## 7. Conclusions and Future Perspectives

Coffee is a preferred beverage globally. Coffee by-products, including CS, SGC, and CH, present valuable sources of health-promoting compounds. The main bioactive components, caffeine, trigonelline, chlorogenic acids, and diterpenoids, have been associated with stimulating brain activity and improving cognitive function. They have potential beneficial effects on the cardiovascular system, the gastrointestinal tract, the immune system, and skin health. Recent research has explored novel strategies to enhance the bioefficacy of these compounds. Systemic bioavailability is essential for maximizing physiological benefits; therefore, evaluating and comparing methods to improve the bioavailability of coffee-derived compounds is critical. This review examined the impact of processing techniques and food matrices on coffee bioavailability. Further studies should investigate the potential of nanotechnology-based delivery systems to protect individual coffee phytochemicals from degradation and to enhance their delivery to the small intestine, thereby increasing bioavailability and bioactivity. This review attempted to find large cohort studies on coffee by-products bioavailability, but evidence remains scarce. Future research needs to focus on human intervention studies for coffee by-products. Human clinical trials involving encapsulated coffee bioactive compounds are recommended. Additional research is required to elucidate the factors that influence individual responses to diets rich in chlorogenic acids from coffee.

## Data Availability

No new data were created or analyzed in this study. Data sharing is not applicable to this article.
